# Oncological and Functional Evaluation of Open Conservation Surgery for Hypopharyngeal Cancer with/without Reconstruction

**DOI:** 10.1155/2018/2132781

**Published:** 2018-03-26

**Authors:** Tsutomu Nomura, Daisuke Maki, Sadahiro Kishishita, Fumihiko Matsumoto, Seiichi Yoshimoto

**Affiliations:** ^1^Department of Otolaryngology, Saitama Medical Center, Saitama Medical University, Saitama, Japan; ^2^Department of Head and Neck Oncology, National Cancer Center Hospital, Tokyo, Japan

## Abstract

**Objectives:**

Oncological and functional results of open conservation surgery for hypopharyngeal cancer have been desired.

**Methods:**

We performed a chart review of 33 patients with hypopharyngeal cancer who underwent open conservation surgery. Oncological and functional results were evaluated in surgery with primary closure (Group A) and surgery with reconstruction (Group B). Postoperative functions were evaluated by interval to resumption of oral intake, Functional Outcome Swallowing Scale (FOSS) and Communication Scale (CS).

**Results:**

Five-year disease-specific and overall cumulative survival rates by Kaplan-Meier method for all cases were 95.7% and 82.3%, respectively. Duration from surgery to full oral intake was 12 days in Group A and 14 days in Group B. FOSS rates were 83.3 in Group A and 95.5 in Group B. CS was 0 in both groups.

**Conclusion:**

Oncological and functional results of open conservation surgery were comparable to those with transoral surgery and chemo/radiotherapy. Our technique represents a reliable treatment for hypopharyngeal cancer.

## 1. Introduction

Hypopharyngeal cancer (HPC) is one of the most aggressive cancers of the head and neck and shows poor prognosis [1]. Most patients already show advanced-stage disease by the time of diagnosis, and the complicated anatomy of this region makes function-preserving treatment difficult. For decades, the standard treatment for tumors involving the hypopharynx has involved radical surgery and postoperative radiotherapy (RT) [2]. Recently, initial surgery has decreased and chemoradiotherapy (CRT) has become the main treatment with the purpose of functional preservation [3]. Although oncological and functional outcomes of CRT are acceptable, with the exception of throat dryness and laryngeal edema, CRT needs a long treatment period (around 2 months or longer) and good renal function and can only be performed once at a single site usually. On the other hand, surgery is becoming more and more limited to radical surgery or early-stage endoscopic and transoral surgery. Transoral robotic surgery [4] and transoral resection [5–7] have recently been introduced and are gaining popularity. Transoral resection is one of the treatments of choice, but achieving good positioning can be difficult and special instruments are needed. Robotic surgery may be superior to other approaches, but is costly, which is problematic in Japan. In our institute, open conservation surgery has been performed for a long time. No previous studies have reported on postoperative function after open conservation surgery. To address the prejudice of poor postoperative function after open surgery, this study evaluated functional and oncological results of cases treated using open conservation surgery.

## 2. Materials and Methods

We performed a chart review of 33 patients with HPC who underwent open conservation surgery in the Department of Head and Neck Oncology at National Cancer Center Hospital (NCC) between July 2007 and December 2012. Each preoperative stage was determined using physical examination, endoscopy, computed tomography (CT), and other examinations with 7th UICC classification [8]. Our criteria for open conservation surgery were as follows. Favorable patient factors include age less than 80 years; performance status of 0 (fully active, able to carry on all predisease performance without restriction) to 1 (restricted in physically strenuous activity but ambulatory and able to carry out work of a light or sedentary nature); good lung function (with normal spirometry and chest X-ray); no history of aspiration; and good prognosis for swallowing rehabilitation (the patients with eating normal food before surgery, good dentition, and having a motivation of eating). Favorable T factors included mobility of bilateral vocal cords and no deep invasion into the arytenoid or postcricoid areas. If the contralateral vocal cord was intact, surgery was indicated despite T3 or T4 status. For the N factor, any status other than N3 was considered favorable. The following surgical procedure was performed for open conservation surgery.

**Figure 1:**
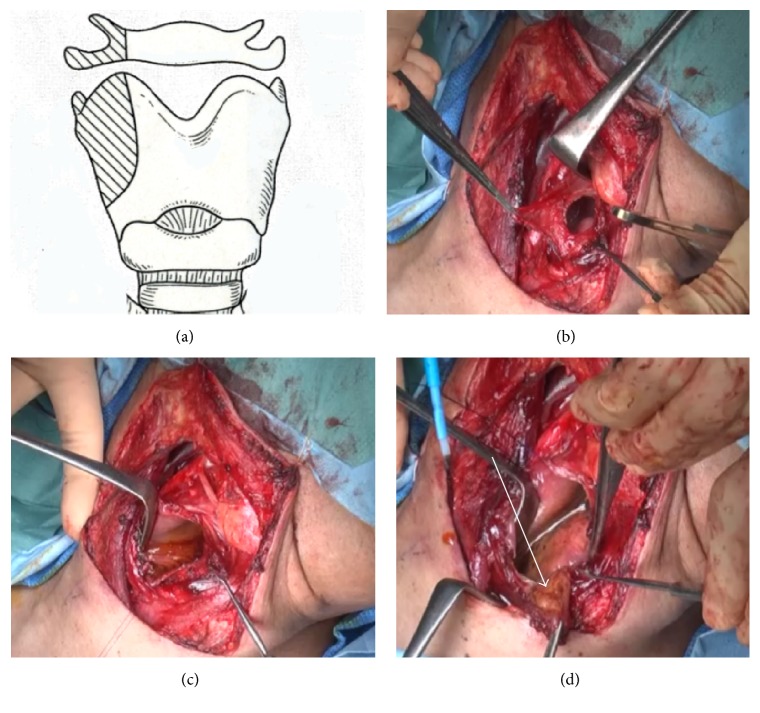
(a) Hyoid bone and laryngeal cartilage were dissected as figure drawings. (b) The tumor was exposed. (c) Lugol's solution (iodine-glycerin) was used. (d) The tumor was resected with more than 5 mm margin.

First, we utilized a suprahyoid or posterior approach depending on the tumor sites (Figure 1). Approach sites is selected carefully not injuring tumors and achieving safety margins. Laryngeal cartilage was dissected as shown in Figure 1(a) in all cases. And hyoid bone was cut as figure in almost of cases.

To check the tumor margins, Lugol's solution (iodine-glycerin) was used (Figure 1(c)). A surgical margin of more than 5 mm was considered safe. To ensure free margins, frozen sections from at least 4 directions were examined histopathologically. Smaller defects were closed primarily, and larger defects were reconstructed using a radial forearm flap or jejunal flap. Patients with open conservation surgery with primary closure (Group A) underwent 1 bilateral and 6 unilateral neck dissections. Patients with open conservation surgery with reconstruction (Group B) underwent 9 bilateral and 12 unilateral neck dissections. Postoperative irradiation was performed in 7 cases from Group B and no cases from Group A. The distribution of all cases is listed in Table 1. Histological examination identified all cases as squamous cell carcinoma (SCC). TN classifications of all cases are listed in Table 2. All patients were followed for a minimum of 2 years or until death. Mean duration of follow-up was 50.0 months.

In all cases, the interval from surgery to resumption of full oral intake was recorded. Postoperative function was evaluated using the Functional Outcome Swallowing Scale (FOSS) criteria [9], 1 year after surgery. Symptom criteria were as follows: stage 0, normal function and asymptomatic; stage 1, normal function with episodic or daily symptoms of dysphagia; stage 2, compensated abnormal function manifesting as significant dietary modifications or prolonged mealtime (without weight loss or aspiration); stage 3, decompensated abnormal function with weight loss of ≦10% of body weight over 6 months caused by dysphagia or daily cough, gagging, or aspiration during meals; stage 4, severely decompensated abnormal function with weight loss of ≥10% of body weight over 6 months caused by dysphagia or severe aspiration with bronchopulmonary complications and nonoral feeding for most nutrition; and stage 5, nonoral feeding for all nutrition.

FOSS rate was counted as the rate of grades 0 and 1 in total cases.

The Communication Scale (CS) was used for evaluating speech function [10]. Symptom criteria were as follows: stage 0, normal speech; stage 1, minimally dysphonic; stage 2, grossly dysphonic; stage 3, near-total loss of speech; stage 4, requiring speech aid; stage 5, no speech. Comparative statistical analysis, overall survival (OS), and disease-specific survival (DSS) rates by Kaplan-Meier analysis and log-rank test were evaluated using SPSS version 19.0 software (SPSS, Chicago, IL).

## 3. Results

### 3.1. Therapeutic Outcomes

Therapeutic outcomes are listed in Table 3. In Group A, mean operation time was 2 h 16 min (range, 1 h 5 min–4 h 30 min), and mean blood loss was 28 ml (range, 2–123 ml). In Group B, mean operation time was 7 h 24 m (range, 5 h 25 min–9 h 35 min), and mean blood loss was 254 ml (range, 2–710 ml).

In reconstructed patients, no flap failure was found.

In terms of oncology, one of the two recurrences in Group A was treated by Endoscopic Mucosal Resection (EMR) and the other in a second open conservation surgery. In Group B, the 4 cases of recurrence were treated by irradiation, and control without recurrence was achieved in 3 of those 4 cases. Five-year DSS rates in Groups A and B were 100% and 92.9%, respectively. OS rates in Groups A and B were 73.3% and 88.4%, respectively. Five-year DSS and OS rates for the total cohort were 95.7% and 82.3%, respectively. There is no statistic difference in two groups.

### 3.2. Functional Analysis

Mean interval from surgery to full oral intake was 12 days (range, 6–862 days) in Group A and 14 days (range, 8–77 days) in Group B. Although the interval to full oral intake was long in one case from Group A because of fistula formation, the mean interval to full oral intake tended to be longer in Group B, although the difference was not significant. In terms of CS score, all cases were classified as grade 0. FOSS rates were 83.3 in Group A and 95.5 in Group B. Functional results tended to be relatively better in Group B than in Group A, but no significant difference was evident.

## 4. Discussion

Evidence as to whether surgery or CRT is more effective against HPSCC is lacking, with the exception of treatment for advanced resectable tumor. The choice of treatment thus depends on the individual institutions. Open conservation surgery offers numerous advantages over transoral resection. No special instruments are needed, and direct observation is possible intraoperatively. The ease of access to the posterior cricoid and caudal regions is also beneficial. If neck dissection is needed, such operations can be performed through the same incision. However, transoral resection carries a risk of fistula formation if neck dissection is undertaken simultaneously. Compared with RT, the very short treatment period and lack of late complications with open surgery are advantageous. Given this background, we compared our oncological and functional results with other modalities of RT and transoral resection as laryngeal preserving modalities.

From an oncological perspective, open conservation surgery had wide range of survival rate [11–28]. Ogura et al. [11] reported the use of partial laryngopharyngectomy (PLP), supracricoid hemilaryngopharyngectomy (SCHLP), and supraglottic hemilaryngopharyngectomy (SGHLP) techniques for early to relatively advanced-stage hypopharynx carcinoma (Table 4). With those techniques, the resection wound underwent primary suturing. With large defects, stenosis of the pharynx and functional loss may also result. After the free flap technique became standard, although small defects underwent primary closure, larger wounds were reconstructed using free flaps as in our technique. No stenosis forms if a free flap is used. In the present study, 5-year OS and DSS rates were 82.3% and 95.7%, representing better results than other open conservation surgeries. Czaja and Gluckman [14] reported a 5-year OS rate of 33.4% for early-stage cancer and commented that surgery with wide resection offered better prognosis than partial pharyngectomy. However, the prognosis depends on the technique and wide resection is not needed if margins are adequate. Compared with transoral resection, few reports have provided survival data, and Tomifuji et al. [6] reported 5-year OS and DSS rates of 77% and 95% for T1 to T3 cases. Such results are excellent and comparable to our own. Reports of CRT and RT seem to skew toward advanced cases. A few reports have showed stage distributions similar to our cases. Mok et al. [29] compared outcomes between intensity-modulated RT (IMRT) and conventional RT. That report described 181 cases in T1 to T4. The 3-year OS rates for IMRT or 3D-RT were 50% and 52%, respectively. Furukawa et al. [30] reported the 5-year OS rate for concurrent chemoradiotherapy (CCRT) after poor response to induction chemotherapy (ICT) was 53.6%, compared to 68.7% with good response to ICT. Nishimura et al. [31] reported a 5-year survival rate of 81% for T1 and T2 hypopharyngeal cases. From these CRT case studies, our 5-year OS of 82.3% was acceptable and relatively better than the data of Nishihara et al. including only T1-2. With a view to improving the results of open conservation surgery, we used frozen sections for intraoperative pathological analysis. This system worked well in our study, and all surgeries were performed safely. The recurrence rate was slightly higher in Group A than in Group B. Four cases of open conservation surgery requiring salvage were able to undergo further function-preserving surgery. Whereas recurrences in RT patients required radical surgery, salvage surgery for open conservation surgery could be performed conservatively. Open conservation surgery thus seems advantageous from the perspective of salvage risk.

In terms of oral intake, all surgical patients were taking food orally within 2 weeks. Open surgeries appear to represent a reliable choice for oral nutrition. Tomifuji et al. [6] reported an interval of 9 days until swallowing after surgery in transoral videolaryngoscopic surgery (TOVS). These data were relatively better than our own, but percutaneous endoscopic gastroscopy (PEG) was necessary in some cases. Tomifuji et al. also reported that 89.8% of patients were less than stage 2 in FOSS. These data were almost the same as our data (Group A, 83.3%; Group B, 95.5%). Our open surgery technique is thus comparable to TOVS from a functional perspective. Few reports have provided functional data for RT and CRT. Recently, many patients have been treated using IMRT. Daly et al. [32] reported the efficacy of IMRT. Szuecs et al. [33] and Peponi et al. [34] reported on swallowing and vocal function after RT, identifying long-term swallowing dysfunction in patients, particularly those on CRT regimens. Although no significant differences were identified, FOSS data tended to be better in Group B than in Group A. Reconstruction surgery takes longer than primary suture, but reconstruction is worthwhile for securing good postoperative function.

## 5. Conclusion

Open conservation surgery does not require special instruments or a long time for treatment with acceptable oncological and functional outcomes. This option is useful in select patients, even in the era of transoral surgery, and should be reevaluated.

## Figures and Tables

**Table 1:** Distribution of patients.

	Cases	Age (years)	Subsite		
			PS	PC	PW
Group A	12	68 (52–79)	8	1	3
Group B	21	65 (51–76)	13		8
(Forearm, 4 cases; jejunum, 17 cases)					

Group A, open conservation surgery with primary closure; Group B, open conservation surgery with reconstruction.

**Table 2:** TN and stage classification.

	Number	N1	N2	Stage	
Group A					
T1	8			I	8
T2	3			II	3
T3	1			III	1
Group B					
T1	1			I	1
T2	9	3	4	II	9
T3	3		1	III	6
				IV	5

Group A, open conservation surgery with primary closure; Group B, open conservation surgery with reconstruction.

**Table 3:** Background for surgery and posttreatment function.

	Time	Blood loss (ml)	OI (days)	CS	FOSS score	Rec	Survival rate: 5-yr	
							DSS	OS
Group A	2 h 16 m	28	12	0	83.3	2/12 (16.7%)	100%	73.3%
Group B	7 h 24 m	254	14	0	95.5	4/21 (19.0%)	92.9%	88.4%
Total						6/33 (18.2%)	95.7%	82.3%

Group A, open larynx conservation surgery with primary closure; Group B, open larynx conservation surgery with reconstruction; OI, interval from surgery to full oral intake; CS, Communication Scale, number of abnormal patients; FOSS, Functional Outcome Swallowing Scale, rate of grade 0 or 1; Rec, recurrence; DSS, disease-specific survival rate; OS, overall survival rate.

**Table 4:** Review of the literature.

	Case	T				Stage				Recon	Survival rate %				Surgery
		1	2	3	4	1	2	3	4		2-y	3-y	5-y DS	5-y OS	
Ogura et al. 1980 [11]	85	9	50	64	52							59.0			PLP
Laccourreye et al. 1993 [12]	34	0	34	0	0	0	21	9	4				55.8		SCHLP
Chevalier et al. 1997 [13]	48	14	34	0	0							52.0		47.0	SGHLP
Czaja and Gluckman 1997 [14]	9					Stage I, II								33.4^*∗*^	
Eckel et al. 2001 [15]	46	T I, II											75.9	61.1	
Makeieff et al. 2004 [16]	87	14	73	0	0	3	36	18	30					60.3	SGHLP
Asakage et al. 2004 [17]	12	2	8	2	0	0	4	3	5	12				50.0^*∗*^	
Plouin-Gaudon et al. 2004 [18]	34	6	25	2	1	1	7	9	17	24			65.0	50.0	
Kania et al. 2005 [19]	147	29	89	16	13	12	39	41	55					54.9	SCHLP
Matsuura et al. 2009 [20]	17	2	6	11	3	2	2	10	9	13			88.9		
Hirano et al. 2010 [21]	14					0	0	2	12	8			66.7	57.1	
Seino et al. 2010 [22]	16	3	8	5	0	2	4	3	7	16	84.0				
Lim et al. 2011 [23]	23	9	14	0	0	5	8	2	8		77.0		61.0		
Joo et al. 2012 [24]	43	0	25	13	5	0	8	11	22	43			67.0	63.0	
Kurita et al. 2012 [25]	32	7	20	3	2	4	12	6	10	32				41.0^*∗*^	
Ono et al. 2012 [26]	15	5	9	1	0	5	5	0	5	1		90.9			
Chung et al. 2013 [27]	58	10	35	7	6	3	10	8	27	58			78.0	77.8	
Kuo et al. 2013 [28]	28	0	22	6	0	0	6	6	16				65.0	48.0	PLP
Our data 2016	33	9	19	5	0	9	12	7	5	21			95.7	82.3	

Recon: reconstruction; OS: overall survival; PLP: partial laryngopharyngectomy; SCHLP: supracricoid hemilaryngopharyngectomy; SGHLP: supraglottic hemilaryngopharyngectomy. ^*∗*^Recalculated by author.
